# Benchmarking of Multi-Modal Partial Discharge Sensors and a Cross-Modal Corroboration Framework for Noise Discrimination in Air-Insulated Medium-Voltage Metal-Clad Switchgear

**DOI:** 10.3390/s26175572

**Published:** 2026-09-02

**Authors:** Tohid Shahsavarian, Ryan D. Sparacino, Mavis Bekoe, Jason Cook, Vincent Tanguay

**Affiliations:** Applied Technology Services (ATS), Pacific Gas and Electric (PG&E), San Ramon, CA 94583, USAmavis.bekoeyarney@pge.com (M.B.); jason.cook@pge.com (J.C.); vincent.tanguay@pge.com (V.T.)

**Keywords:** partial discharge, medium-voltage switchgear, noise discrimination, acoustic emission, cross-modal corroboration, PRPD, multi-modal sensing, wideband electromagnetic

## Abstract

Reliable partial discharge (PD) assessment of air-insulated medium voltage (MV) metal-clad switchgear is challenged by the close proximity of adjacent units and the noise environment generated by control, relay, and mechanical or electromechanical equipment within the substation. Combined sensing approaches have been employed to identify primary PD sources and distinguish them from interference signals. Although individual sensing technologies have been extensively studied, no systematic framework has been established to corroborate readings across sensing modalities under controlled, reproducible noise conditions. This study presents a laboratory benchmark performed on an actual 15 kV metal-clad switchgear assembly, incorporating representative PD source configurations and interferences encompassing electrical, acoustic, and electromagnetic noise. Sensors spanning multiple detection classes were evaluated, including acoustic sensors (intrusive and remote airborne, and surface-contact types), high-frequency current transformers (HFCTs), transient earth voltage (TEV) sensors, and a wideband electromagnetic sensor, across both open-access and closed-panel configurations. Testing showed that electrical and electromagnetic noise primarily affects conducted-electrical and radiated-EM channels, while acoustic noise degrades acoustic sensors, leaving each channel type largely unaffected by the other’s interference. This complementary behavior supports a two-path corroboration criterion that reduces false positives and false negatives and informs practical sensor selection for field deployment.

## 1. Introduction

MV metal-clad switchgear serves as the primary interconnection between the utility distribution grid and the feeders that supply electricity to customers. Ensuring the uninterrupted operation of these apparatuses is essential to maintain grid stability and prevent costly downtime [1,2]. Unplanned switchgear outages can lead to cascading failures and costly maintenance interventions for grid operators and utilities. The main root cause of switchgear failure is insulation degradation, including localized partial discharge and dielectric breakdown where the electric field exceeds the dielectric strength of the insulation covering the metallic parts. PD activity typically precedes catastrophic insulation failure and can continue for months to years, a process that can be accelerated by harsh, uncontrolled environmental conditions [3]. Early PD detection and characterization is therefore a key element of proactive monitoring to avoid these consequences [4,5,6].

IEC 60270 [7] has been the main established reference for conventional, charge-calibrated PD measurement of current pulses for each discharge event, enabling traceable and quantitative analysis. However, operational substations rarely allow the opportunity to de-energize the circuit and deploy the calibrated coupling capacitors as required by this standard. Research attention has consequently shifted toward online, non-intrusive PD diagnostics that use alternative sensor modalities to exploit different physical coupling pathways between the PD source and transducers. Three broad families of unconventional methods, specified by CIGRE Technical Brochure 662 and IEC TS 62478 [8,9], include high-frequency current transformer (HFCT) sensing, acoustic and ultrasonic detection, and electromagnetic detection, including HF, VHF and UHF couplers and antennas. The sensitivity, noise immunity, and practical deployability of each of these sensing categories determine its suitability for different applications, equipment types, and operating conditions.

A PD plasma channel radiates mechanical waves and transmits pressure impulses through the insulating medium and metallic enclosures, which can be detected by acoustic sensors [10]. Contact-based piezoelectric probes are capable of detecting structure-borne ultrasonic waves through the outer structure of enclosed compartments, at the cost of substantial attenuation through the metal walls [11]. Airborne ultrasonic waves escaping from access ports, vents, or gaps can be captured by sensors inserted into these areas or swept around accessible apertures. Parabolic reflectors with either single or multi-microphone beamforming arrays are variations of ultrasonic PD detectors; these can image acoustic emission from exposed, open-panel equipment insulation from a distance and provide location data without physical contact [12,13]. The rate of change of electromagnetic fields coupled to the grounded enclosure, generating traveling transient voltage pulses along the metallic enclosure, can be measured via TEV probes [14,15]. On the other hand, high-frequency current pulses travelling on the ground path can be measured with HFCT sensors clamped onto earth straps or cable screens [16,17]. The intensity of each of these signals depends on the type of partial discharge and the medium transmitting and coupling the emitted waves through air and structures. Broadband electromagnetic waves from a PD source can be detected by wideband or UHF antennas in either an attached position (where conducted energy is measured and the metal enclosure acts as a large parasitic antenna with strong signal and conducted noise sources) or a remote position through free air for open-panel, exposed, energized insulated components, which offers substantially better noise immunity [18,19]. The breadth of available sensing modalities, each with distinct sensitivity, coupling pathway, and noise immunity characteristics, provides users with a versatile toolkit; however, the nature of the PD source and the site-specific electrical, electromagnetic, or acoustic noise environment ultimately dictate which modalities hold a practical advantage.

Several research groups have performed multi-modal PD detection evaluation on MV switchgear. Reid et al. [14] aimed to establish empirical pulse-by-pulse relationships between TEV, IEC 60270, and UHF responses for well-controlled laboratory discharge sources. They demonstrated that no universal theoretical conversion factor exists between techniques because of their inherently different, source-dependent behaviors. Alvarez Gomez et al. [20] characterized and compared UHF antenna and HFCT sensing methods for online PD monitoring in MV metal-clad switchgear with designed internal void and surface discharges in a laboratory environment. They showed that combined deployment yields greater diagnostic capability, with UHF offering autonomous defect-type identification and HFCT providing superior sensitivity and detection of PD activity near the inception voltage. Hussain et al. [21] evaluated a differential electric field sensor (D-dot) and a loop antenna installed inside the compartment, along with a Rogowski coil and HFCT, confirming that all responded to the same physical discharge activity and ranking the D-dot sensor as the most suitable for online monitoring based on its combination of size, cost, ease of installation, and signal to noise ratio. Lachance and Rosa [22] compared conventional IEC 60270, stand-alone TEV sensor and ultrasonic camera with a clear line of sight on assembled 15 kV and 35 kV metal-clad switchgear. They reported that the TEV sensor captured discharges of high apparent charge, that the camera was effective for external defects, and that conventional measurement with a coupling capacitor remained the most sensitive and reliable option. Zhang et al. [23] proposed a distributed TEV wireless sensor network with a two-step approach that uses a support vector machine (SVM) classifier for pattern recognition. Ewaida et al. [24] applied the cumulative energy method (CEM) to estimate the onset time of each TEV signal and the time difference of arrival (TDOA) to locate the PD source. Machine learning approaches have also been proposed to automate PD source identification and pattern recognition in MV switchgear [25,26,27]. Barrios et al. [28] proposed a convolutional autoencoder trained on continuous wavelet transforms to classify four real defect types found in in-service switchgear, including cable termination faults, with deep-learning de-noising. Chang and Lin [29] trained convolutional, residual and lightweight networks on phase-resolved and pulse-sequence patterns to recognise six defect types across gas-insulated switchgear and cable joints, raising accuracy from 86.9% to 97.5% with a noise-trained ensemble of the three models. They also report that wavelet filtering removes white and out-of-band noise but leaves pulse-type interference, such as corona, power-electronic switching and communication carriers, indistinguishable from genuine discharge.

Despite this progress in understanding individual sensor modalities, a unified, field-ready diagnostic framework is still missing. First, no prior study has simultaneously assessed a full suite of acoustic, HFCT, TEV, and wideband EM sensors; published comparisons are limited to two or three modalities [14,20,21,22]. Second, although electromagnetic and acoustic noise is widely cited as the primary obstacle to reliable field PD investigation [7,9,30], the characteristics of the different noise types and their interaction with each sensor’s coupling pathway have not been fully mapped in the literature. Third, no operationally deployable cross-sensor discrimination and validation protocol has been derived from multi-sensor data covering a full set of discharge-geometry and noise-overlay conditions. Without such a protocol, field operators have no systematic basis for determining whether an elevated sensor reading is due to genuine PD activity or to the local noise environment. This paper addresses all three gaps through a systematic laboratory evaluation on a full-scale MV metal-clad switchgear test bench.

## 2. Test Bench Design and Experimental Methodology

### 2.1. Switchgear Test Platform and Sensor Deployment

**Figure 1:**
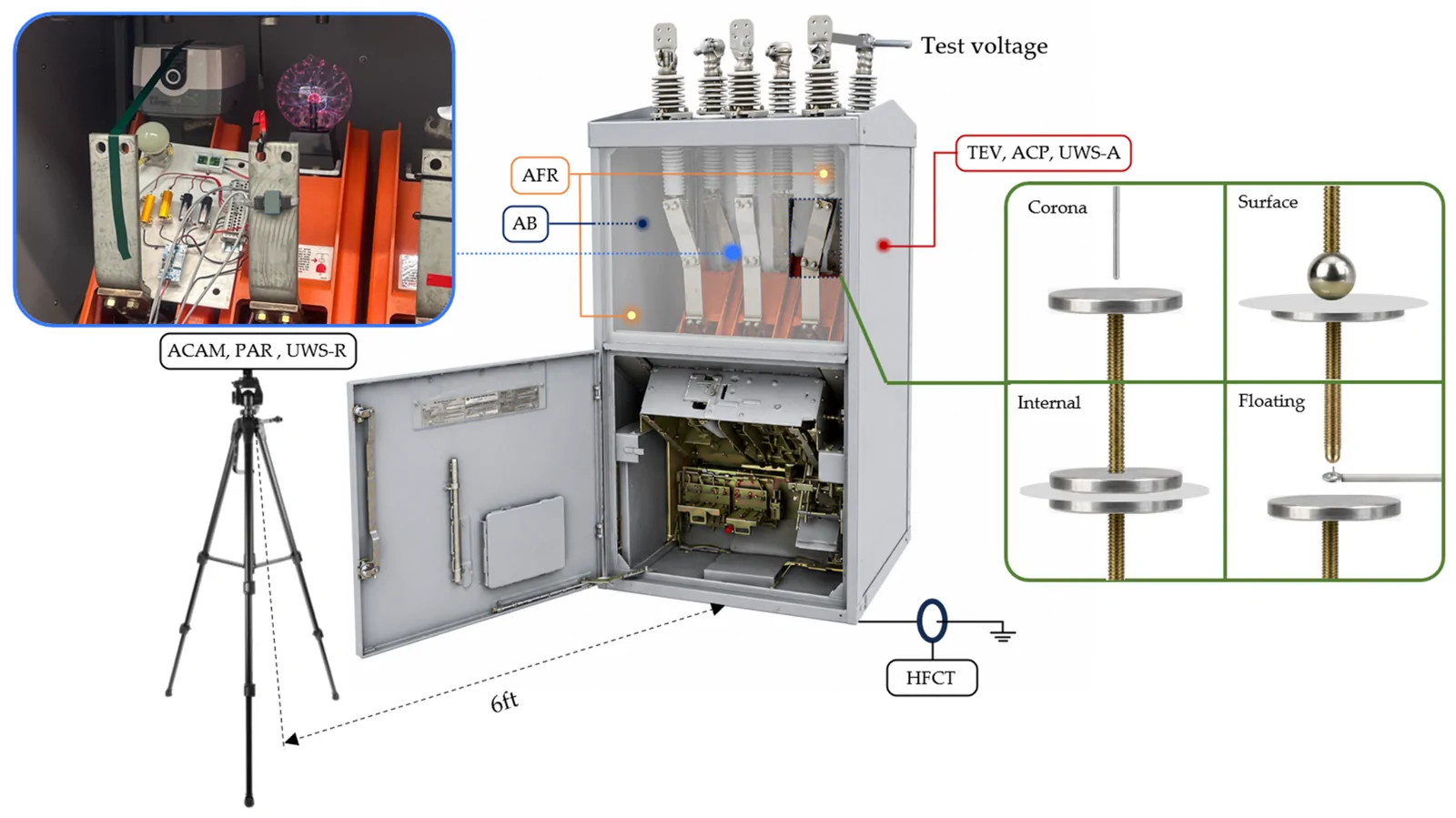
Switchgear 3D laser scan with sensor, PD source, and noise source locations.

The laboratory test platform consists of a 15 kV, 1200 A rated outdoor metal-clad distribution switchgear (GE, Burlington, IA, USA) with vacuum interrupters held in the closed position, verified PD-free, and confirmed to be within the manufacturer’s specified vacuum pressure tolerance prior to testing. Figure 1 presents a three-dimensional laser scan of the switchgear, acquired using a HandySCAN 3D MAX handheld laser scanner (FARO CREAFORM AMETEK, Lake Mary, FL, USA), indicating the deployment positions of all sensing elements investigated in this study.

Measurements were conducted under two panel configurations to reflect the range of access conditions encountered in field deployments. Under the open-panel configuration, and in compliance with minimum safe working distance requirements applicable to energized equipment in substation environments, an acoustic camera (ACAM), parabolic acoustic receiver (PAR), and ultra-wideband electromagnetic sensor (UWS) were deployed at a standoff distance of 6 ft (1.8 m) from the switchgear. The same ultra-wideband sensor was used in both configurations and is distinguished by position throughout: UWS-R at the 6 ft remote standoff with the panel open, and UWS-A attached to the panel surface. Readings from the two positions are reported separately and are not averaged. The UWS is a self-contained instrument whose electric-field antenna couples capacitively to the field radiated by the discharge current, requiring no galvanic connection to the energized assembly. Its response is flat in amplitude and phase from 0.5 MHz to an internal cut-off at 50 MHz, with a manufacturer bench sensitivity of about 10 mV/pC measured at 1 cm from a reference source, which is not transferable to the standoff geometries used here. Each pulse is digitized at 200 MS/s and 8-bit resolution through a programmable 0 to 40 dB input amplifier, and its full waveform, timestamp and phase angle are recorded, the phase reference being taken from the same antenna sensing the supply-voltage field on the component under test. Only an amplitude threshold was applied to reject the noise floor; the waveform clustering and automatic defect classification available in the instrument were disabled, so that all sensors were compared on the same basic quantities of pulse magnitude and phase. Under the closed-panel configuration, the remaining sensors were applied directly to the enclosure surface. As indicated in Figure 1, the sensor complement is organized into four categories: (i) UWS-A, acoustic contact probe (ACP), and transient earth voltage (TEV) sensors mounted at fixed positions on the panel surface; (ii) an acoustic bolt (AB) sensor inserted into the panel; (iii) an airborne flexible receiver (AFR) positioned toward two open bolt holes on the front panel; and (iv) HFCT sensors clamped onto the ground strap. Conducted PD magnitudes measured by the HFCT are reported throughout as $q_{HFCT}$, the magnitude displayed by the handheld instrument in pC. No charge calibrator was injected into the switchgear under test, so $q_{HFCT}$ is an indicated instrument reading, not apparent charge traceable to IEC 60270. It is used for comparison between test conditions in this study and is not directly comparable to apparent charge values reported by other systems. To support corroboration of sensor readings and assess inter-unit variability, two independent sets of sensors were procured from different manufacturers for each modality.

A dedicated reference measurement circuit, comprising a calibrated 1 nF coupling capacitor and an HFCT connected to a data acquisition unit in accordance with IEC 60270, was installed to establish the background noise floor, and determine the partial discharge inception voltage (PDIV) for each discharge source configuration. Readings from this circuit are not included in the sensor comparison of Section 4 and Section 5, and its phase-resolved PD (PRPD) patterns are not reported here. Conventional charge-calibrated measurement is not the subject of this study, and the signatures of the four canonical geometries reproduced in the test bench are well established in the literature. The calibrated noise floor of the reference circuit was confirmed to be below 2 pC. Ambient temperature and relative humidity inside the switchgear enclosure varied across test sessions in the ranges of 15–20 °C and 48–61% RH, respectively. As environmental effects on PD activity are outside the scope of this study, PDIV and discharge intensity were verified against the reference circuit prior to each data collection session to confirm stable and reproducible discharge conditions.

### 2.2. Partial Discharge Source Simulation

To accommodate the PD test objects within the switchgear enclosure, the interconnecting pad mount between the lower terminal of the feedthrough bushing and the circuit breaker terminal was removed, exposing the high-voltage connection point. Four canonical PD source geometries, corresponding to different defect types encountered in service, were constructed and installed at the location indicated in Figure 1. The electrode configuration and dimensions of each geometry are described below.

Corona discharge: A 2 mm diameter aluminum rod with a hemispherical tip served as the high-voltage electrode, positioned with its tip 10 mm from the surface of a grounded aluminum flat disc with smooth, rounded edges to eliminate incidental field enhancement at the disc perimeter.

Surface discharge: An aluminum sphere acting as the high-voltage electrode was placed in contact with a 2.54 mm thick circular PTFE dielectric film (diameter: 130 mm), which rested on a grounded aluminum flat disc of 100 mm diameter.

Internal void discharge: Three layers of 2.54 mm thick PTFE film, each 130 mm in diameter, were stacked and sandwiched between two flat plate electrodes. A 2 mm diameter cylindrical void was created at the center of the middle film. To prevent the onset of surface discharge at the triple junction region surrounding the upper energized electrode, that area was filled with a silicone encapsulant with a relative permittivity of 2.9 and a volume resistivity of 4.5 × 10^13^ Ω·cm.

Floating electrode discharge: A metallic washer with an outer diameter of 17 mm, with attached small upward protrusion on its upper face, was suspended 2 mm from a 10 mm diameter energized brass rod by a horizontal dielectric support arm fixed to the switchgear wall, leaving the washer electrically floating. A grounded disc electrode was positioned 28 mm from the axis of the high-voltage rod and 17 mm from the center of the washer.

### 2.3. Noise Source Circuit Design

Real substation environments present a wide range of electrical, acoustic, and electromagnetic interferences, and assessing each sensing modality under these conditions is central to determining its field readiness. Five noise-generating circuits were therefore constructed and installed inside the switchgear at the positions shown in Figure 1, selected to represent disturbance sources commonly found in close proximity to distribution switchgear. Each circuit couples energy into the measurement environment through a distinct physical pathway (conducted current, radiated electromagnetic fields, or airborne and structure-borne acoustic pressure), allowing the coupling selectivity of each sensor type to be characterized independently.

Phase-Controlled AC Load Controller (Phase-cut AC): This circuit uses a thyristor-based variable speed controller to drive a 100 W incandescent halogen lamp from a 120 V/60 Hz mains supply.

High-Frequency Switched-Mode Power Supply (SMPS): A PULS QS5.241 industrial DIN-rail 24 V DC supply feeds an LM2596 step-down converter driving a 12 Ω wire-wound resistive load at approximately 37.8 W continuous dissipation. The LM2596 switching frequency is fixed at 150 kHz, injecting a narrowband conducted noise train at that frequency and its harmonics.

Electronic LED Driver: A Hatch RL12-60M-LED electronic transformer is a 30 W constant-voltage AC/DC switching converter with an internal isolation transformer. In the designed circuit for this study, it is loaded with a 5 Ω wire-wound resistor in place of the lamp. Its internal topology combines a line-frequency rectifier with a 30–60 kHz resonant high-frequency inverter; residual 120 Hz mains ripple on the DC bus modulates the high-frequency carrier.

Narrowband (NB) Ultrasonic Source: A benchtop stainless-steel ultrasonic cleaning tank was placed inside the enclosure with its piezoelectric transducer bonded to the tank base driven at 42 kHz. Energy radiates both through the tank walls as structure-borne vibration and into the surrounding air as a narrowband acoustic pressure field at the transducer frequency.

External Corona Discharge Source (Plasma ball): A Discovery plasma globe, powered from a 12 V DC adapter, contains a Tesla-coil resonant transformer that raises the supply to several kilovolts at 20–50 kHz to sustain a visible plasma discharge within the gas-filled sphere.

### 2.4. Measurement Repeatability

Each measurement was acquired three times under identical conditions, and the mean of the three acquisitions is the value reported. For the four modalities measured with two instruments (PAR, ACP, AFR and TEV), the reported value is the mean across both instruments, so each entry combines six acquisitions.

For every sensor and every test condition the standard deviation of the three acquisitions, $S_{i}$, was calculated. For example, readings of 28.4, 29.2 and 29.9 dB give $S_{i}$ = 0.75 dB. Each sensor was characterized at n such conditions, between 17 and 74, covering the voltage steps, the noise overlays and the de-energized baseline. These were combined into a single repeatability standard deviation for that sensor by pooling the variances,

(1) $$S_{e}=\sqrt{\frac{\sum S_{i}^{2}}{n}}$$

which is the scatter to be expected of any single reading from that sensor. Expressing each $S_{i}$ relative to its own mean and averaging over the *n* conditions gives the coefficient of variation,

(2) $$CV=(\sum \frac{S_{i}}{{\bar{x}}_{i}})\times \frac{100}{n}$$

where ${\bar{x}}_{i}$ is the mean of the three acquisitions at condition *i*. Decibel readings were converted to a linear amplitude scale before applying (2), since a ratio formed from decibel values depends on the reference level; the reference itself cancels in the ratio.

The two instruments of a paired modality also differ from one another by a fixed offset. The standard deviation of that offset, $S_{b}$, was obtained from the differences between the two instruments’ means at each condition, after subtracting the contribution of acquisition noise. The uncertainty of a reported value (*u*) then depends on how many acquisitions and instruments it averages,

(3) $$u_{1}=\frac{S_{e}}{\sqrt{3}}\;(\mathrm{one}\;\mathrm{instrument})$$

(4) $$u_{2}=\sqrt{\frac{S_{b}^{2}}{2}+\frac{S_{e}^{2}}{6}}\;(\mathrm{two}\;\mathrm{instruments})$$

Table 1 lists these quantities for every sensor. A single instrument gives no estimate of its own offset, so *u* for the sensors deployed as one unit describes repeatability alone and excludes any difference a second unit of the same type would introduce. Relative repeatability ranges from 4.4% for the attached wideband sensor to 18.4% for the transient earth voltage probes, the conducted and electromagnetic channels repeating more closely than the acoustic ones. Within a given sensor, individual conditions vary more widely; for the TEV probes $S_{i}$ ranges from 0.2 to 4.2 dB, the largest values occurring for the floating-electrode source, whose intermittent charge and spark-over cycle gives less stable readings than the other geometries. For all four paired modalities $S_{b}$ exceeds $S_{e}$, by factors between 1.1 and 4.8, so the choice of instrument was a larger source of variability than the repeatability of the reading. The values of *u* are used in Section 6.2 to identify which observations in this study are resolved by the measurements and which are not.

## 3. Baseline Noise Source Characterization

Each noise source simulated in this study replicates common types of electrical, electromagnetic, and acoustic disturbances encountered in operational substations. These interferences can readily produce false positives (or mask genuine PD pulses) when using single-modality PD monitoring systems. While partial discharge events and noise sources each exhibit distinct temporal, spectral, and coupling characteristics, predominantly affecting either electrical, electromagnetic, or acoustic pathways, the most challenging scenarios occur when a noise source couples energy through the same physical mechanism as the PD activity. Table 2 summarizes realistic substation equipment and systems that correspond to each simulated noise source.

**Figure 2:**
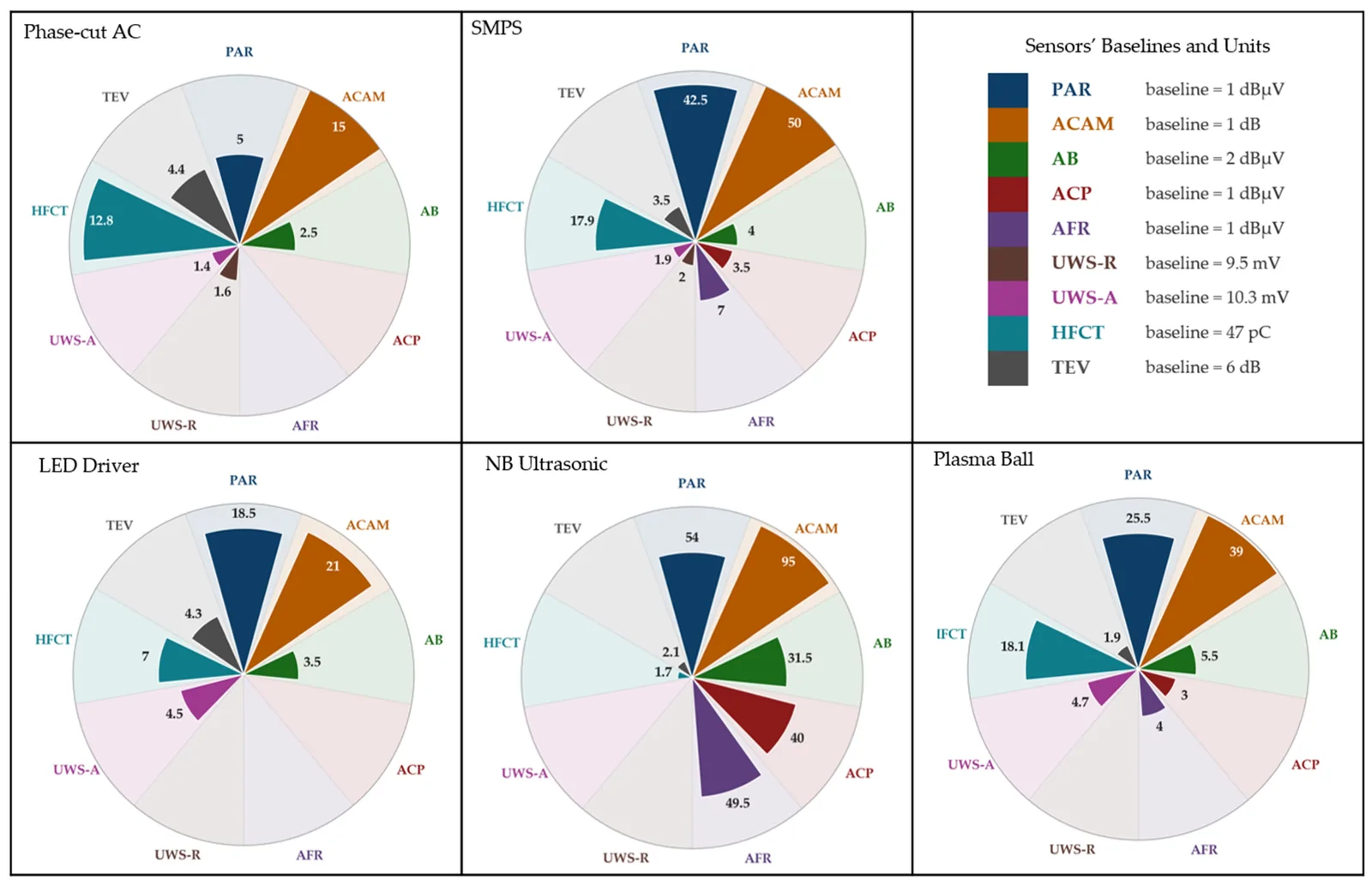
Sensor responses to the five noise sources at baseline (de-energized).

Noise sources were activated individually prior to energizing the switchgear and before introducing any PD sources. Figure 2 presents polar bar charts showing each sensor’s response to each noise source. Measured values for each sensor are normalized to that sensor’s quiescent ambient levels obtained in the laboratory, as indicated in the legend. Since the switchgear was not energized during the test session, all measurements were performed with the front panel removed, allowing the use of all sensors positioned at various locations as shown in Figure 1. Key observations from these graphs along with each noise source’s coupling physics are summarized below.

### 3.1. Phase-Cut AC

Thyristor switching produces fast current transients on the order of 100 ns, phase-locked to the power frequency, injecting high-frequency pulses onto the ground return where HFCT is installed. A secondary effect is emission of radiated broadband electromagnetic interferences (EMI), moderately detected by TEV. PAR and ACAM, both of which have exposed analog front-ends, are the most susceptible to this radiated EMI coupling into their electronics. ACAM is particularly affected due to its large microphone beamforming array, which increases its sensitivity to incident electromagnetic fields from noise sources. Consequently, ACAM (15× baseline) and HFCT (12.8× baseline) exhibit the highest noise susceptibility. With the exception of ACP and AFR, which remain at their baselines, the other sensors show a mild to moderate response to this noise. UWS-A and UWS-R both showed negligible response: although the transient edge carries energy into the lower part of the sensor’s band, the radiated field reaching either position is too weak to lift the reading above its quiescent level.

### 3.2. High-Frequency Switched-Mode Power Supply (SMPS)

The buck converter’s continuous 150 kHz switching produces harmonic noise on the ground conductor, broadband radiated electromagnetic fields, and magnetostrictive vibration from the inductor core. This results in a relatively strong, simultaneous excitation across all sensor modalities. HFCT responses reached 17.9× their quiescent baseline, while ACAM and PAR exhibited the highest acoustic susceptibility, peaking at 50× and 42.5×, respectively. All acoustic sensors showed consistent, moderate sensitivity to this noise, and the TEV sensor behaved similarly to its response under the previous noise source. UWS, however, was minimally affected, as the harmonic content does not extend sufficiently into its operating frequency band.

### 3.3. Electronic LED Driver (30–60 kHz)

This AC/DC switching converter, which incorporates an internal isolation transformer, injects conducted high-frequency noise onto the switchgear’s metallic structure and ground path. However, due to the driver’s certified EMI filtering and its galvanically isolated transformer, the conducted noise is less detectable than that produced by the SMPS and phase-cut AC sources. Unlike the LM2596 SMPS module used here, with exposed PCB circuitry and an unenclosed inductor, the Hatch driver is a fully enclosed commercial unit with a compact metal housing that partially damps the magnetostrictive vibration and suppresses ultrasonic emission. As a result, AFR and ACP show no measurable interference, whereas AB remains sensitive enough to measure a 3.5× increase over baseline. Under the same noise conditions, the Hatch driver produces a stronger near-field signal at the UWS in the attached position, likely due to its direct connection to the main bus, in contrast to the SMPS, which is powered from a 24 V DC DIN-rail supply located outside the enclosure. Nevertheless, no significant radiated EM field is generated to excite the UWS in the remote location. PAR and ACAM remain strongly affected by all noise sources for the same reasons described for the phase-cut AC noise source.

### 3.4. Narrowband Ultrasonic Acoustic Source (42 kHz)

The piezoelectric transducer inside the ultrasonic cleaner generates narrowband 42 kHz ultrasonic waves that couple directly into the switchgear enclosure, saturating all acoustic channels while producing negligible electromagnetic emission. The induced vibration is strong enough to excite even the ACP sensor. The device operates at a fixed frequency with a continuous sinusoidal drive rather than as a pulsed source, and with the switchgear de-energized it produced no measurable response on the HFCT, so no discrete high-frequency current is coupled onto the ground path. No significant EM radiation is produced within the operating band of the UWS either, leaving it unaffected. Section 5.1 notes one condition in which the conducted and enclosure-coupled channels do rise while this source is active.

### 3.5. External Corona Discharge Source

The plasma generator used in this study is a miniature Tesla-coil device with a centrally driven high-voltage electrode operating at approximately 35 kHz and 2–5 kV. The coil ionizes the noble-gas fill and sustains continuous plasma filaments, producing high-frequency current transients on the ground conductor and generating audible and ultrasonic crackling from partial arcs inside the globe. These emissions couple into both the conducted electrical path and the acoustic path. Unlike the other noise sources, the elevated readings from PAR and ACAM here are predominantly due to genuine ultrasonic waves. Although corona discharge is known to emit electromagnetic radiation, the UWS in the remote position does not capture these signals, consistent with its behavior under all other noise sources. Whether this is due to fundamental differences between the plasma in this device and classical corona (such as its continuous-wave nature versus corona’s discrete pulse events, or the confinement of the discharge within a sealed glass sphere), or simply the UWS’s limited sensitivity to EM radiation at the 6 ft remote standoff distance, will be addressed by evaluating the sensor under a pure corona discharge in the next section.

## 4. Partial Discharge Detection Performance Under Clean Condition

The previous section presented the designed noise sources and the coupling mechanisms through which their conducted and radiated emissions interact with different sensing modalities, enabling an assessment of how field noise affects each sensor. This section now evaluates the capability of the various sensors to detect well-known PD sources and characterizes their responses in a noise-free environment. Descriptions and geometric details of each PD source are provided in Section 2. Table 3 reports the sensor readings at the voltage at which each PD source initiates discharge (PDIV) and at several higher voltage steps, up to the point where audible activity becomes noticeable to personnel. Because the UWS and HFCT sensors exhibit substantially higher measurement ranges, their results are presented in a separate panel using logarithmic color scaling for the cell values. Measurements for UWS-R, PAR, and ACAM were obtained with the front panel removed and the sensors positioned 6 ft away. For all other sensors, data were collected with the front panel installed, in accordance with the safety protocols required when approaching the switchgear.

### 4.1. Surface Discharge

This type of discharge develops along the interface between a solid dielectric and the surrounding air when the tangential component of the electric field exceeds the surface withstand. It represents surface tracking and creepage phenomena that occur on contaminated or aged insulation, for example, pollution or moisture accumulation on bushings, insulators, spacers, and cable terminations.

Table 3 illustrates the strong responses obtained from sensors operating across different modalities. Conducted PD pulses traveling through the ground strap reach 307 pC at PDIV and increase to 2014 pC at three times PDIV. Electromagnetically coupled PD activity on the metalwork is detectable by both TEV and UWS. On the acoustic side, airborne emissions are readily captured by AFR and AB at close range, and by PAR and ACAM at the remote position. However, the coupled acoustic signal is not sufficiently strong for ACP to detect with high confidence relative to the other sensors. UWS also weakly detects this discharge at the remote position, and its PRPD gives a complementary indication of surface discharge in noise-free condition.

### 4.2. Floating Discharge

This discharge occurs when a metallic component is electrically isolated, i.e., bonded neither to ground nor to the energized conductor. The component charges capacitively, and a spark-over occurs once the potential across the adjacent gap exceeds the air breakdown voltage determined by the gap distance. At higher applied voltages, the charge–discharge cycle accelerates, producing more intense PD activity. Measurements were obtained only at PDIV and 1.5×PDIV, as the intermediate voltage levels did not yield sufficiently distinctive readings, and 1.5×PDIV was high enough for the resulting metal vibration and discharge to be audibly detected.

At inception, the floating-electrode source drives the electrical and electromagnetic channels far harder than surface discharge, with the widest gap on the UWS and HFCT channels, while the airborne acoustic readings remain at comparable levels. The reason lies in how the discharge forms, not in the higher inception voltage. An isolated metal element charges capacitively until its gap breaks down, then releases almost all of its stored charge in a single fast metal-to-metal spark. That event sends a large charge through a steep, fast-rising current pulse, which the HFCT measures on the ground conductor as 1300 pC at inception against 307 pC for surface discharge. Since the spark path is metallic and connects straight to the enclosure and ground, its high-frequency transient couples efficiently into the conducted and electromagnetic channels (HFCT, TEV, and UWS). Surface discharge works the other way. It begins at a lower voltage as many small creeping events, and part of that charge stays bound on the dielectric surface, so its electrical signature grows only as the applied voltage rises.

### 4.3. Corona Discharge

Corona is a gas discharge that forms in air around sharp, energized conductors when the local electric field exceeds the ionization threshold without fully bridging the electrode gap. Each event carries a small charge but occurs at a high repetition rate, so it radiates ultrasonic and audible energy efficiently. It typically arises at points and protrusions on energized hardware exposed to air, such as poorly dressed conductor ends or sharp corners on busbars and connectors. The result is a source that is acoustically loud but produces little electrical signal. All acoustic-based sensors show their highest readings for this PD source, while the HFCT and the attached UWS give their lowest responses of the four sources. This also answers the question left open in Section 3 about whether the UWS can detect corona: it can, but the EM emissions from corona are weaker than those from the other PD mechanisms.

### 4.4. Internal Discharge

Internal or cavity discharge occurs inside a gas-filled pocket enclosed within solid insulation and ignites when the local field across the void exceeds its breakdown threshold. Because the discharge is confined within the dielectric, its acoustic and EM emissions are heavily shielded by the surrounding solid material. The test object, three stacked PTFE layers with a void at the center, reproduces the voids and delamination found in solid insulation such as cast-resin bushings, spacers, epoxy components, and aged cable insulation. It is one of the most damaging defect types, since it degrades the bulk insulation from within and is the hardest to detect from outside the enclosure.

Because the emission is buried in the insulation, the ACP stays fully blind up to 1.25 times PDIV, and the AFR responds weakly at PDIV and only improves at higher voltages. The remote acoustic sensors respond more clearly, although their readings for the void remain among the weakest they produce across the four sources. The electrical and EM channels remain effective: the high-frequency conducted current pulse escapes the void and travels through the metallic structure far more readily than the acoustic wave, so the HFCT, TEV, and attached UWS pick up the conducted signal reliably.

## 5. Sensor Response Under Combined Discharge and Disturbance Conditions

Section 3 and Section 4 characterized the sensing modalities separately: first against the noise sources alone, then against each PD source alone. In the field the two occur together, and this section evaluates that superposition. Each PD source is energized while every noise source is applied in turn, so the sensors can be judged under the combined conditions they face in service. The tests were run in the closed-panel configuration, with the front panel installed. ACAM, PAR and UWS-R require the front panel to be removed, which is not permissible on energized switchgear during routine inspection, so they are not included in the combined tests. Their intrinsic response to each discharge geometry and their susceptibility to each noise source are reported in Section 3 and Section 4. A further limitation applies to these three: viewing the cubicle from one fixed external position, they cannot separate a discharge from an interferer when both are located inside it. Section 5.1, Section 5.2, Section 5.3 and Section 5.4 therefore cover the sensors usable with the panel closed, which is the condition under which the proposed two-path corroboration criterion would be applied in service. To gauge each remaining sensor’s sensitivity against the active noise, every PD source was driven at its PDIV. The figure for each geometry is a set of polar charts, one per sensor, in which the five wedges are the five noise overlays. Each wedge carries three normalized bars (noise only, PD at PDIV only, and the two superimposed shown in yellow, light red, and dark red, respectively). Values are normalized to each sensor’s maximum reading across all tests for that specific PD source. The relative heights of the three bars within each wedge carry the diagnostic information, and several cases warrant particular attention:

- Good noise immunity shows as a small yellow bar with the light-red and dark-red bars close together: the noise adds little, and superimposing it barely changes the PD reading.
- When the yellow bar matches or exceeds the light-red bar, the noise alone produces as much signal as the discharge, so the combined reading is noise-dominated and any indication it gives is a likely false positive.
- When the dark-red bar rises above the light-red bar, the noise adds to the genuine PD reading and inflates the apparent discharge level, an over-read that can be mistaken for more severe activity than is present.

### 5.1. Surface Discharge Under Superimposed Noise

**Figure 3:**
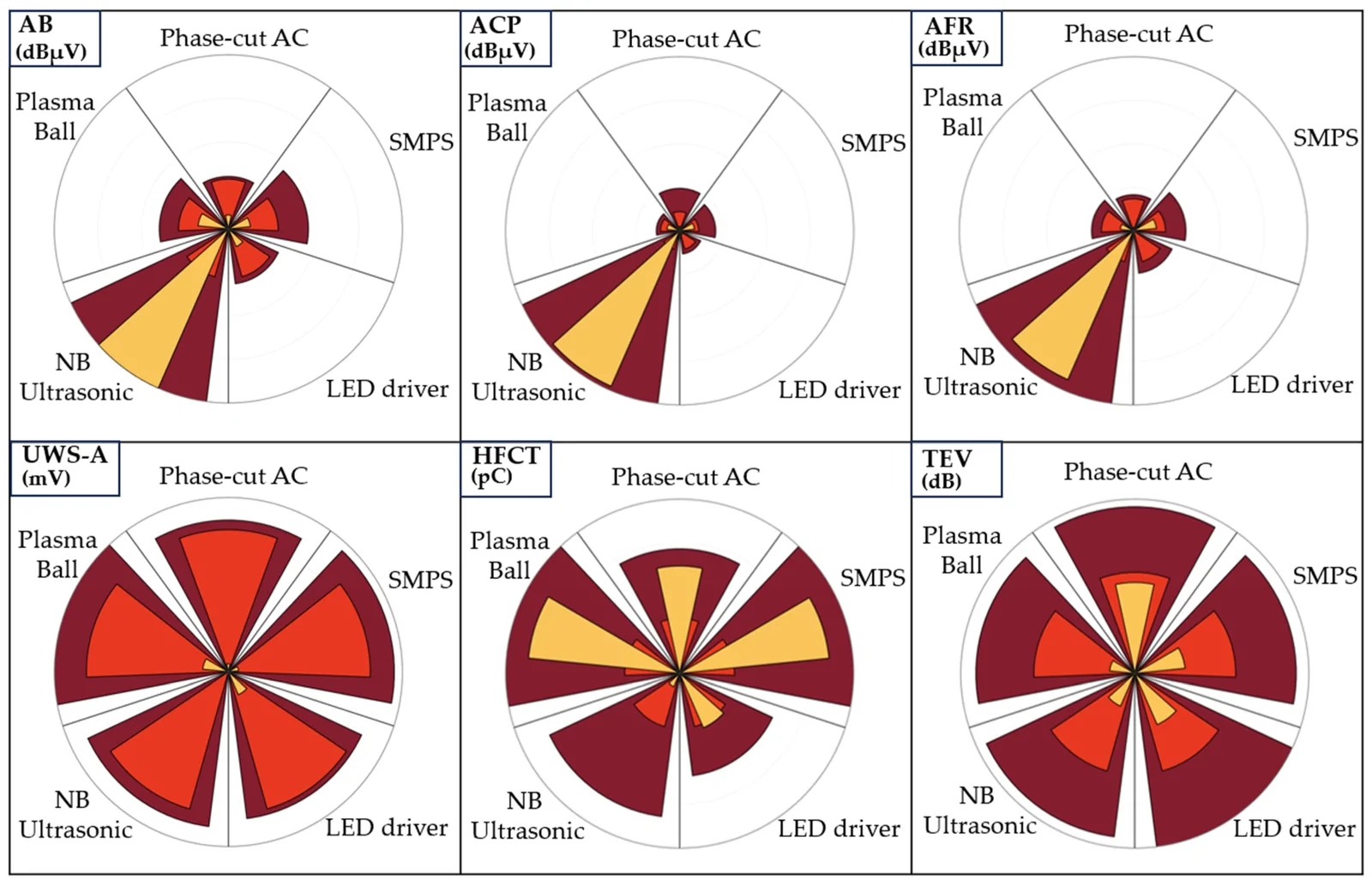
Sensor responses to surface discharge under each superimposed noise source: noise only (yellow), PD at PDIV (light red), and both superimposed (dark red).

Surface discharge was detected reasonably well by every modality, but how much of each sensor’s capacity is usable depends on the noise level, because this geometry begins at a lower inception voltage than the other sources. At PDIV its conducted signal is comparatively modest (307 pC), while the electrical noise sources alone drive the HFCT to 330–850 pC, which can blur the distinction between discharge and interference pulses, as shown in Figure 3. This becomes less of a concern once the surface discharge has progressed to a more severe state, since higher applied voltages produce markedly greater discharge intensity that overshadows the noise. The TEV reading likewise rises as the discharge intensifies, but it is comparatively more immune to electrical interference, so its combined response is cleaner than the HFCT’s, although under the phase-cut AC source the noise alone (18 dB) approaches the 20 dB discharge reading. The same holds for the attached wideband sensor, which captures the discharge effectively even under superimposed noise; its combined reading sits at or slightly above the clean-discharge level, because the electrical and electromagnetic interferers add little within its band and the acoustic overlay does not couple into it, so the response stays well above the noise floor. Among the acoustic sensors, the intrusive bolt performs best, owing to its direct acoustic access to the enclosure interior.

### 5.2. Floating Discharge Under Superimposed Noise

**Figure 4:**
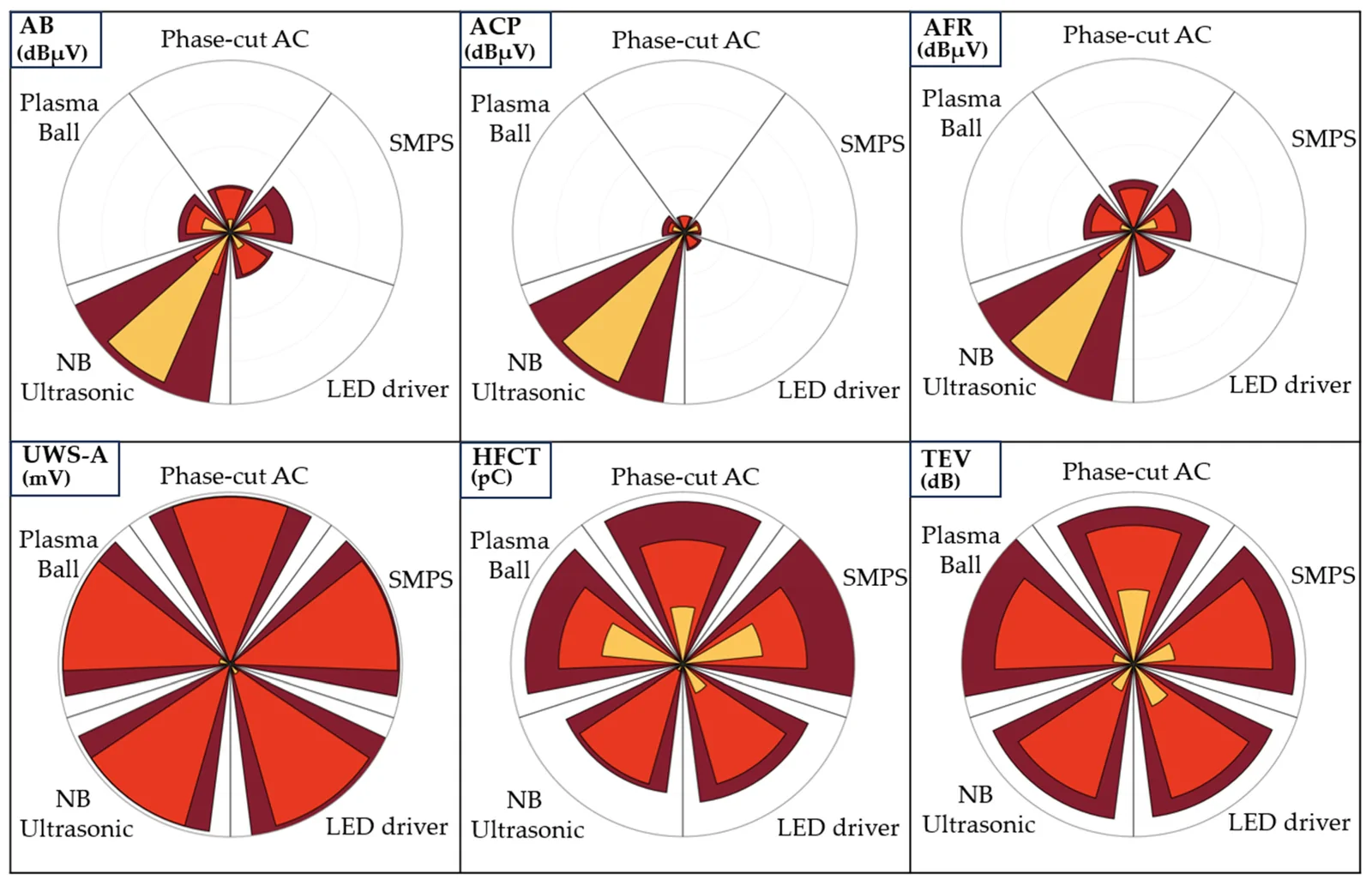
Sensor responses to floating discharge under each superimposed noise source: noise only (yellow), PD at PDIV (light red), and both superimposed (dark red).

Floating discharge was identified as the strongest electrical source of the set, giving the largest conducted magnitude and the strongest transient earth voltage. This strength is visible in Figure 4, where the clean-discharge bar stands well above the noise-only reading for every interference source, so the combined bar is governed by the discharge activity. The HFCT ranks second: the floating-discharge pulses travelling along the ground path (1300 pC) exceed every noise-only reading (up to 850 pC), even though the interference still inflates its combined output. Corona and floating discharges face the same interferers; however, the outcome diverges entirely. The floating discharge has enough inherent strength to keep the electrical channels reliable, whereas corona, as shown next, does not. The attached UWS holds its reading across every overlay: the combined magnitude lands on or just above the clean-discharge level and stays far above the noise-only contribution, and the PRPD pattern, obtained with only the amplitude threshold, can provide complementary confirmation once the magnitude criterion has been met. The acoustic sensors also captured the discharge above the noise level, with the single caveat of the NB ultrasonic source, which remains the dominant interferer for this modality.

### 5.3. Corona Discharge Under Superimposed Noise

**Figure 5:**
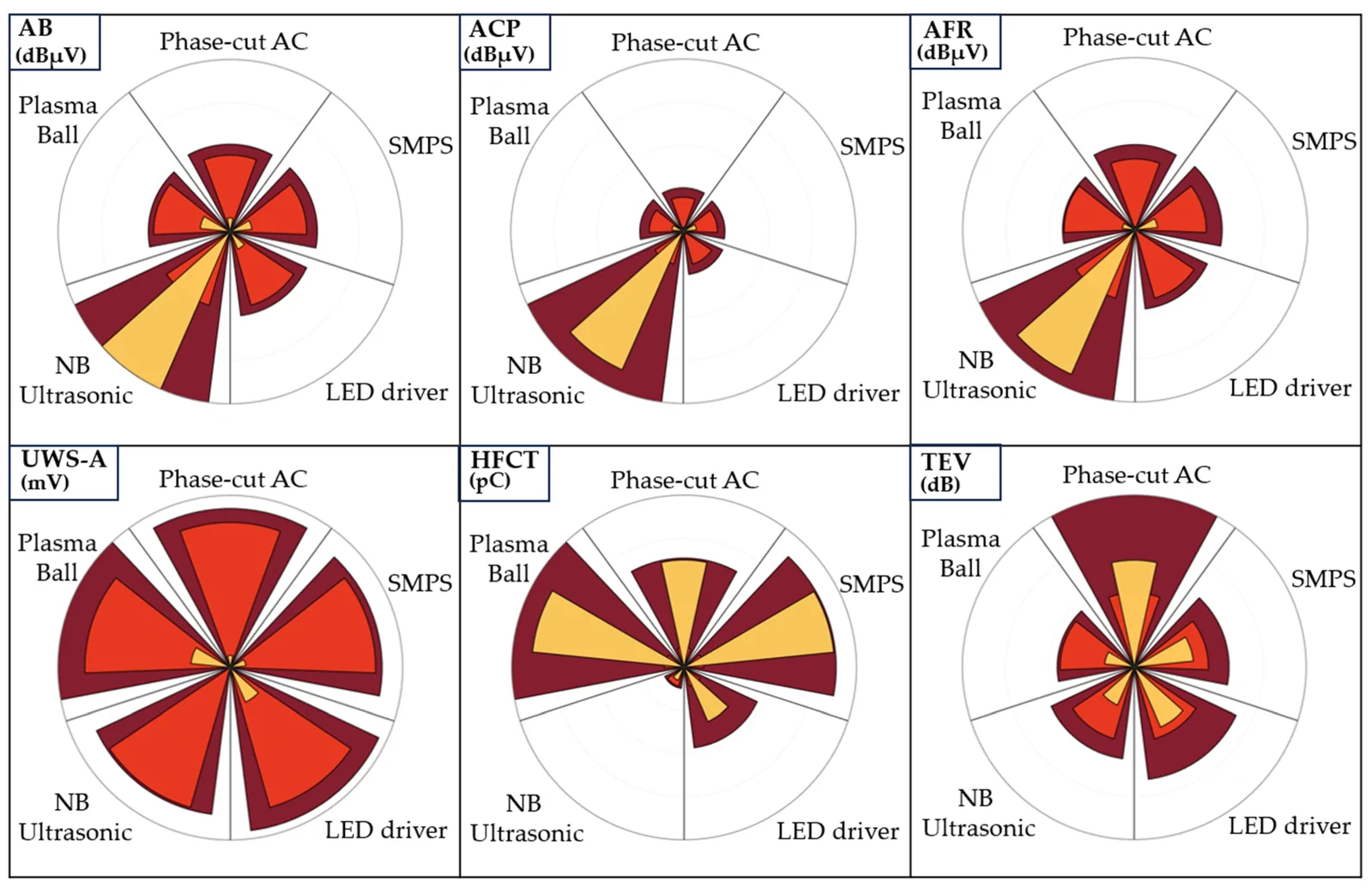
Sensor responses to corona discharge under each superimposed noise source: noise only (yellow), PD at PDIV (light red), and both superimposed (dark red).

Section 4 showed corona discharge as acoustically loud but electrically faint, producing the smallest conducted signal of the four geometries while producing strong airborne ultrasonic waves readily detectable by sensors in this category. The high-frequency transient currents that most noise sources, except the NB ultrasonic, inject into the ground path exceed the corona signal itself (330–850 pC of noise against a 110 pC discharge), so the HFCT reading is dominated by interference. The TEV and the attached UWS were less affected by these sources, although the TEV’s inherently low corona response (12.5 dB) leaves little margin: under the phase-cut AC overlay the noise alone (18 dB) exceeds the clean discharge reading. Readings from the acoustic sensors are far more dependable: none of the electrical interferers radiate strong acoustic energy compared with the corona source, so the clean and superimposed bars for AB, ACP, and AFR sit almost on top of each other, well above the noise level (see Figure 5). The single exception is the ultrasonic noise source, whose narrowband field saturates every acoustic sensor. Under this specific condition, the HFCT, which does not transduce acoustic energy, provides the more reliable indication.

### 5.4. Internal Discharge Under Superimposed Noise

**Figure 6:**
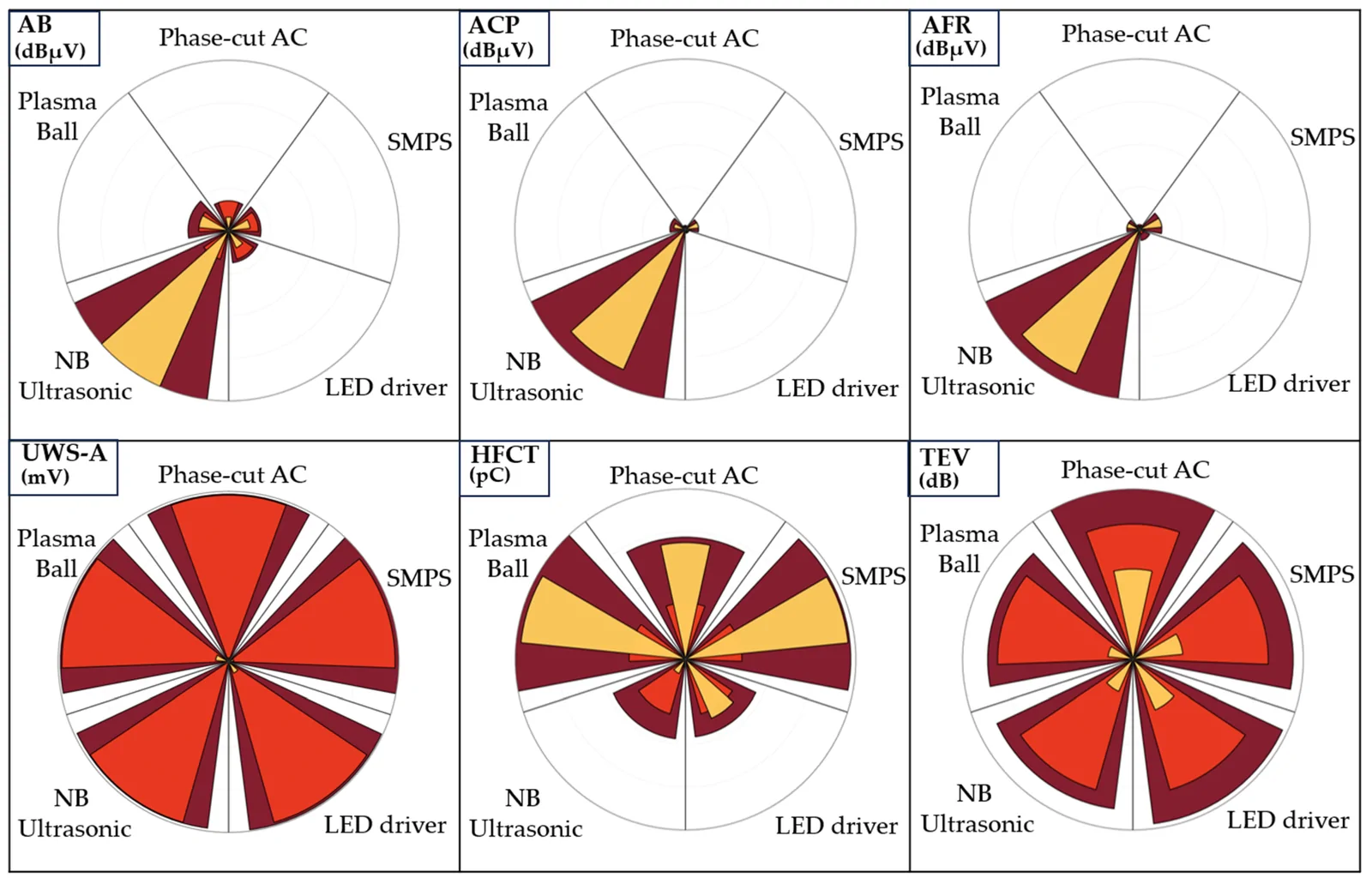
Sensor responses to internal discharge under each superimposed noise source: noise only (yellow), PD at PDIV (light red), and both superimposed (dark red).

**Figure 7:**
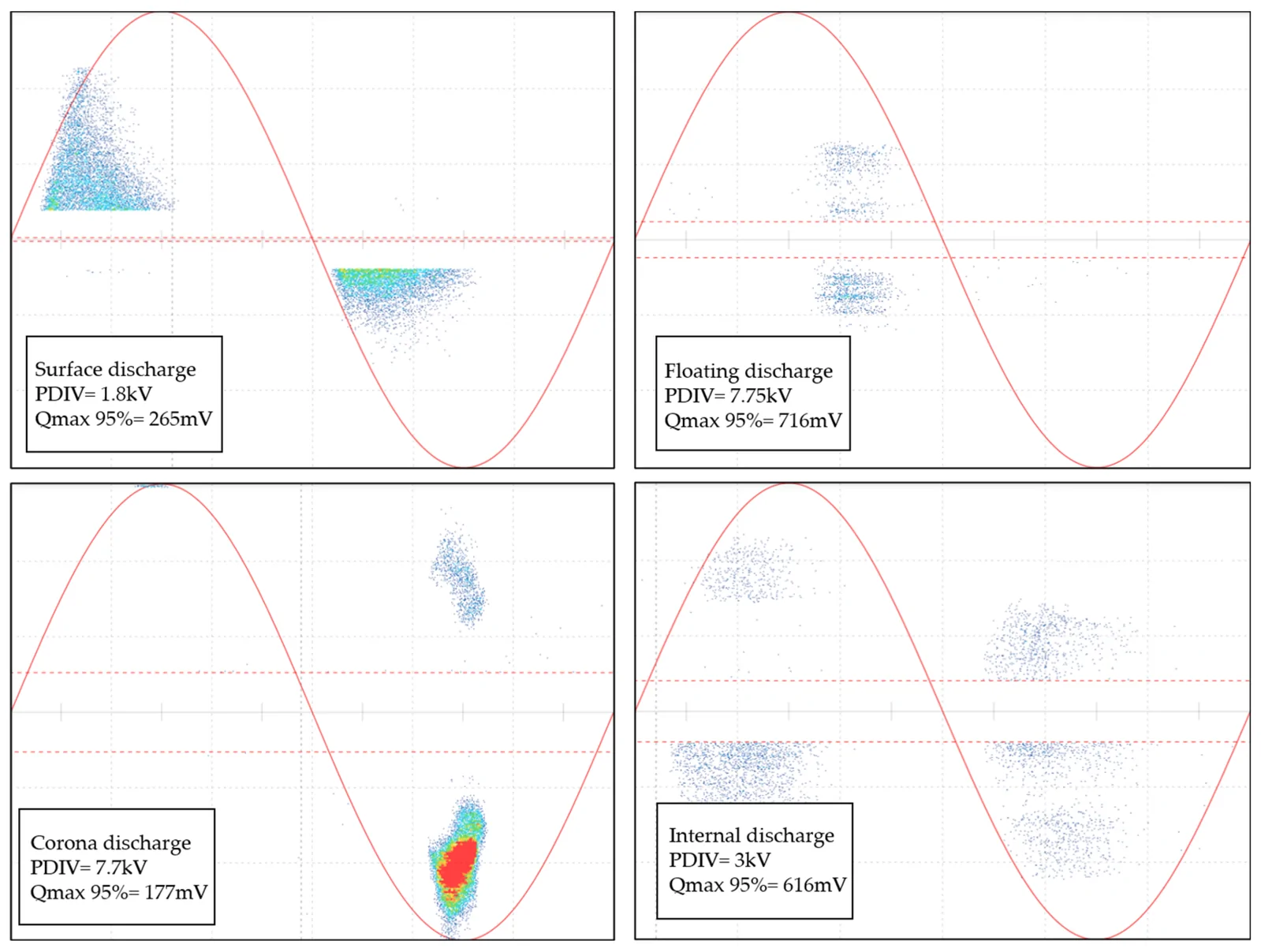
PRPD patterns from the attached wideband sensor (UWS-A) at each defect’s inception voltage: the red curve is the applied voltage, the dashed lines mark the amplitude threshold, and point color indicates pulse density from blue (low) to red (high).

Section 4 identified the internal void as the most concealed of the four geometries, since the discharge is trapped within the solid insulation and its emissions must cross both the dielectric and the enclosure wall before reaching any external sensor. TEV is the strongest performer here. Its combined bars remain large and consistent across all five noise overlays, sitting close to the clean discharge reading and well above the noise contribution, which confirms its established role as an internal-discharge detector and shows that the noise sources do not mask the void signal it captures on the enclosure (See Figure 6). The HFCT also detects the void, as the conducted pulse escapes along the ground path, but at inception the electrical noise sources alone produce 330–850 pC against the 290 pC discharge signal, so its indication is noise-dominated until the activity intensifies. The attached wideband sensor performs much like the electrical channels here. It captures the void strongly in clean condition and keeps that reading under every noise source: the combined bars settle on or just above the clean-discharge level while the noise-only contribution stays small, so the channel over-reads marginally instead of losing the signal. Among the acoustic sensors, only AB genuinely identifies the void, owing to its direct access to the internal air space, and it holds against the electrical interferers with two caveats: the plasma-ball noise alone reaches the same 11 dBμV as the void reading at inception, and the NB ultrasonic source saturates it as before. The internal discharge is therefore confirmed most dependably by the TEV on the electrical side, by the attached wideband sensor on the electromagnetic side, and by the intrusive bolt on the acoustic side, with the HFCT corroborating once the discharge margin exceeds the conducted noise. The attached UWS reading is further supported by its clear PRPD patterns, as shown in Figure 7, that remain distinct once low-magnitude events are excluded by the amplitude threshold, although the evaluation in Section 6.2 rests on magnitude alone. Table 4 provides the complete set of sensor readings used to generate the bar plots presented in Section 5.1, Section 5.2, Section 5.3 and Section 5.4.

## 6. Discussion, Cross-Modal Corroboration Framework and Decision Support

The previous sections examined how the discharge and noise sources behave individually and how their combined presence affects the sensitivity and reliability of each sensing modality. Each sensor was examined first on its own response to each defect type and then under realistic interference, which exposed where a reading can be trusted and where it can mislead. This section draws those observations together into a more instructive and procedural form. It compares the modalities against one another, condenses their performance into a single matrix, and sets out how to approach an unknown field condition and how to interpret and corroborate the measurements taken on site.

### 6.1. Sensor Capability by Modality

Conducted electrical signals are the current pulses that travel along the ground path and can be measured against IEC 60270 [7]. Of the sensors studied, the HFCT belongs to this category [16]. It detected all four PD sources, most strongly for floating discharge (1300 pC at inception) and least strongly for corona (110 pC). Most of the noise sources drove it to 330–850 pC with no discharge present, magnitudes comparable to the PD pulses themselves, so the channel is only as trustworthy as the margin between the two. Once the discharge is intense enough to dominate the noise, the HFCT provides good accuracy, and the accompanying PRPD pattern can be examined as complementary information to identify the discharge type [17,30]. The sensor is also largely immune to acoustic noise, so in an acoustically loud environment it can corroborate the acoustic sensors and confirm whether their elevated readings come from a genuine discharge or from interference.

Electromagnetic emission detectors such as TEV and UWS work on different principles and over different bandwidths, yet both provide useful information. TEV detects the transient voltage that a discharge drives onto the enclosure and needs no internal access. It performs well on surface and floating discharge and gives a comparatively low corona response. Although TEV has a higher quiescent baseline than the other contact probes, its noise response generally stays below the detected PD signal (corona, with its low TEV reading, being the exception), and it can benefit from additional post-processing algorithms [15,23]. The attached UWS offers the strongest detection of any sensor relative to its quiescent baseline under noise-free conditions, and it kept that reading in the combined tests: for all four discharge types, its response under each overlay stayed at or slightly above the clean-discharge level rather than being pulled down by the interference. The residual over-read is small relative to the discharge, and the resolved patterns separate it cleanly from the genuine signal once the amplitude threshold is applied. The remote UWS, viewed from a fixed open-panel position and excluded from the combined tests, remains the more exposed of the two to a dense interference train.

Acoustic sensors show strong immunity to most electrical and electromagnetic noise, the exception being an environment that itself radiates energy in the ultrasonic band. Within this group the AB is clearly superior, since it has direct access to the internal compartment air. The AFR also performs well where an access port is available on the equipment panel. The ACP, by contrast, gives weaker readings overall, and both the ACP and the AFR are blind to the internal void at inception; the AFR begins to respond only as the voltage rises.

### 6.2. Cross-Modal Corroboration Framework and Field Decision Support

The performance matrix in Table 5 condenses the observations of Section 4 and Section 5 into a single reference. Each detection rating is the response at PDIV expressed as its elevation above the quiescent baseline in units of the standard uncertainty, u, given in Table 1, and each immunity rating reflects how the reading held when the corresponding noise source was superimposed at PDIV. Expressing both on the uncertainty scale makes the ratings reproducible from Table 1, Table 3 and Table 4, and places detection and interference on a common footing. Read row-wise, the matrix states what an individual sensor can and cannot be trusted to report; read column-wise, it identifies which sensors remain dependable for a given defect type or noise environment.

The matrix shows that no single channel is dependable across all test conditions. The attached wideband sensor is the only one rated good for every discharge geometry, and its interference response is the smallest of any channel, at most 4.2 standard uncertainties. The HFCT gives the largest conducted response in the study, 41 standard uncertainties on the floating electrode, yet it over-reads under every noise source and is the weakest detector of corona.

The acoustic bolt is the strongest acoustic channel and rates good on three of the four geometries, but the narrowband ultrasonic source raises its reading by 47 standard uncertainties, roughly twice its own best detection margin. The acoustic contact probe and the airborne receiver do not respond to the internal void at all: their readings at inception are 0 and 0.2 standard uncertainties above baseline, in agreement with Section 4.4. The margins reported in this section are taken at the inception voltage, where the discharge is least intense and detection is hardest. They increase as the discharge intensifies at higher applied voltage, as the voltage steps of Table 3 show. The ratings in Table 5 and the decision rule evaluated in Table 6 therefore describe the least favorable condition a survey will encounter.

#### 6.2.1. Domain Selectivity

Each kind of interference affects mainly the sensors that respond to that form of energy. The conducted and radiated interferers raise the HFCT by 11 and 28 standard uncertainties and the TEV by 4 to 6, while leaving the airborne receiver within 2 in every case. The narrowband ultrasonic field inverts the pattern, raising the three acoustic channels by 16 to 47 standard uncertainties while the TEV and the attached wideband sensor move by less than 5. The separation follows from the transduction mechanism of each sensor class: a piezoelectric element does not couple to high-frequency current on the ground strap, and a current transformer clamped on that strap does not respond to airborne pressure. The five interferers act through three coupling pathways: conducted ground-return current, radiated electromagnetic field, and airborne or structure-borne acoustic pressure; any further disturbance encountered in service acts through one or more of the same three.

Two of the five sources cross this boundary. The switched-mode supply raises the acoustic bolt by 10 standard uncertainties through magnetostrictive vibration of its inductor, and the plasma source by 6 through genuine acoustic emission from its partial arcs, while both also act strongly on the conducted channel. Interference is therefore predominantly, domain-selective, and a corroboration rule built on the separation must be qualified accordingly.

#### 6.2.2. The Two-Path Corroboration Criterion

A reading should be accepted as genuine partial discharge only when it is corroborated simultaneously by an acoustic channel and by an electrical or electromagnetic channel with demonstrated noise immunity. The qualification matters. As Table 5 shows, the HFCT responds to every interferer at a level comparable with or exceeding a genuine discharge, so admitting any electrical channel would weaken the rule rather than strengthening it. In this study the channels that satisfy the immunity condition are the TEV probe and the attached wideband sensor.

#### 6.2.3. Quantitative Evaluation of the Criterion

The criterion and the single-channel alternatives were evaluated against the measurements, with the results in Table 6. Twenty-four conditions in which a discharge was present, comprising the four geometries at inception both alone and with each of the five interferers superimposed, provide the positive cases; the five de-energized noise-only conditions provide the negative cases. A channel is taken to respond when its reading exceeds its quiescent baseline by k standard uncertainties, and the evaluation is repeated for k from 1 to 5 to show that the outcome does not depend on where the detection threshold is placed.

The HFCT used alone never exceeds 20% specificity at any threshold: four of the five interferers drive it past the detection criterion with no discharge present, so it identifies every discharge but cannot distinguish one from interference. Pooling channels within a domain does not help, because a logical union inherits the false indications of all its members and can be no better than its weakest one; the rule that fires when any electrical or electromagnetic channel responds has exactly the specificity of the HFCT alone at every threshold, and the acoustic union tracks the acoustic bolt. Requiring both domains raises specificity from 20% to 40% at k = 3, since the two families respond to different interferers, but the unqualified rule still reaches only 60% at its best. Excluding the HFCT gives 100% sensitivity and specificity at every threshold of three standard uncertainties or above, because the two sides fail to different interferers: the phase-cut source reaches the electrical side but not the acoustic, and the other four the reverse. The attached wideband sensor achieves the same performance on its own from k = 3, being the only channel with a threshold that separates every discharge in this study from every interferer.

Two limitations apply to these figures. The specificity rests on five noise-only conditions, so a specificity of 100% carries a 95% confidence interval of 57 to 100%; the sensitivity, based on 24 conditions, carries an interval of 86 to 100%. More importantly, the rule was derived from the same measurements against which it is evaluated, so this assesses internal consistency only. Confirmation requires application to equipment whose defect condition has been established by other means, which is discussed in Section 6.3.

#### 6.2.4. Qualifications

The internal void is a legitimate exception on the acoustic side. Its emission is shielded by the solid dielectric, so only the intrusive bolt responds at inception, at 8.2 standard uncertainties, while the contact probe and airborne receiver remain at the baseline. Where no intrusive access exists, a sustained indication on the electrical and electromagnetic channels, with the TEV and attached wideband sensor in agreement, and supported where available by a consistent PRPD pattern, should be treated as a probable internal defect; demanding acoustic corroboration for a buried void invites a false negative on the most damaging defect type. The converse case is an ultrasonically loud environment. The narrowband interferer raised every acoustic channel by 16 to 47 standard uncertainties while leaving the TEV and wideband sensor within 5, so acoustic readings carry no evidential weight there, and confirmation must rest on the electrical and electromagnetic side until the acoustic disturbance is located and silenced.

#### 6.2.5. Defect Identification

The pattern of agreement across modalities also points to the defect type. In these tests, a strong conducted and electromagnetic response with no acoustic activity indicated an internal void; a simultaneous surge on the electrical channels, led by the largest conducted and TEV magnitudes of the test series, a floating defect; a loud acoustic response paired with the weakest conducted signal; corona on exposed hardware; and a moderate response on all channels growing steadily with applied voltage and surface tracking. Phase-resolved patterns sharpen the classification further, since the interferers examined here are either continuous or locked to the supply phase in a manner distinct from the phase-correlated clusters of genuine discharge.

#### 6.2.6. Survey Sequence

Each available channel is compared against its own quiet baseline, and any elevation exceeding three standard uncertainties is classified by domain. An indication confined to the electrical and electromagnetic channels calls for the acoustic channels to arbitrate: if they stay silent and the phase pattern matches the signatures of Section 3, interference is the likely cause, with the internal void as the noted exception. An indication confined to the acoustic channels in an ultrasonically active bay is treated with the same suspicion, and the HFCT, insensitive to acoustic energy, arbitrates in the opposite direction. When a noise-immune electrical or electromagnetic channel and an acoustic channel respond together, the reading is accepted; a discharge-like phase-resolved pattern, where the instrument provides one, adds further confidence. The modality signature is then used to infer the defect type, and repeated surveys establish the trend on which maintenance decisions rest.

### 6.3. Limitations and Scope of Validity

The measurements reported here were made on one assembly under controlled conditions, and the resulting limitations are outlined below.

- Field application: The laboratory setting allowed each discharge and each interferer to be introduced and removed under control, so a given response could be attributed to a specific source and coupling pathway. Field conditions offer no such control and applying the rule during routine surveys, where the interference is unfamiliar, the defect condition unknown, and the available channels limited by access, would establish how well the strategy performs outside the laboratory.
- Source position and distance: The influence of the sensor-to-source distance was not examined systematically. It should matter less for the conducted and enclosure-coupled channels, since the HFCT measures current on the ground path while the TEV and attached wideband sensors couple to the enclosure, so their response follows the coupling route rather than a line of sight to the source. The acoustic channels are the ones where path length and intervening metalwork would reduce the margin. Repeating the matrix with the source displaced within the compartment, and in adjacent cubicles, would quantify this and would show how far the corroboration approach retains its detection accuracy as the source moves away from the sensors.
- Interference coverage: Interferers were applied one at a time, whereas a substation presents several simultaneously, and how the individual margins of Table 5 combine was not tested. The five circuits span the three coupling pathways available to any disturbance, so a further source acts through a pathway already represented; a wider library would add a greater range of energy and repetition rate, such as the high-energy sporadic events produced by switching operations. Interference above the passband of the sensors used here, for example emissions from wireless equipment, cannot reach these channels and would be relevant only for couplers operating at higher frequencies.
- Other equipment designs: One 15 kV outdoor metal-clad design was tested. Since enclosure geometry and ground-strap routing govern acoustic attenuation and transient earth voltage propagation, repeating the benchmark on other designs would establish how far the rankings of Table 5 transfer, as distinct from the pathway separation on which the framework itself rests.
- Independent validation: As noted in Section 6.2, the decision rule was evaluated against the measurements from which it was derived. Applying it unchanged to new cases, in which the presence or absence of a discharge is established by other means, would show whether the performance of Table 6 holds outside the dataset that produced it.

## 7. Conclusions

This study benchmarked nine partial discharge sensing configurations spanning the acoustic, conducted-electrical, and electromagnetic domains on a full-scale 15 kV metal-clad switchgear assembly in the laboratory, against four canonical discharge geometries and five substation-representative noise sources, applied individually and superimposed. To the authors’ knowledge, it is the first evaluation to combine a full multi-modal sensor suite, a characterized noise library, and superimposed discharge-plus-noise testing on the same assembly, addressing the three gaps identified in the introduction.

The results support three main findings. Detection capability is strongly source-dependent: the floating electrode drove the conducted channel to its largest response, the buried void was captured almost exclusively by the electrical and electromagnetic channels, and corona showed the opposite trend, saturating the acoustic sensors while producing the smallest conducted signal; the sensor that is most sensitive in a clean environment is therefore not necessarily the most dependable one once noise is present. Noise vulnerability is predominantly domain-selective: the electrical interferers inflated or masked the HFCT reading at inception-level discharge and affected the TEV moderately, while the attached UWS held its reading nearly unchanged, and the acoustic interferer saturated every acoustic sensor while leaving the electromagnetic channels within five standard uncertainties. Two sources cross the boundary, the switched-mode supply and the plasma ball, both of which reach the acoustic bolt as well as the conducted channel, so the corroboration rule is built on a separation that is strong rather than absolute.

For field practice, a reading should be accepted as genuine discharge only when an electrical or electromagnetic channel and an acoustic channel respond together, subject to the two exceptions documented in Section 6: a buried void may legitimately lack acoustic support, and an ultrasonically loud environment invalidates acoustic confirmation. A minimum field complement follows the same logic: one conducted or electromagnetic sensor with demonstrated noise immunity (TEV or an attached wideband sensor with phase-resolved analysis), paired with one acoustic sensor chosen by access, the intrusive bolt where a port exists and the airborne receiver otherwise. The performance matrix and survey sequence of Section 6 turn these results into a repeatable procedure for separating discharge from disturbance.

## Figures and Tables

**Table 1:** Measurement repeatability and uncertainty.

Sensor	n	$S_{e}$	CV	$S_{b}$	u
One instrument					
HFCT	37	52.9 pC *	4.7%		30.5 pC
UWS-A	37	24.5 mV	4.4%		14.2 mV
UWS-R	17	5.6 mV	6.3%		3.3 mV
AB	37	1.91 dBμV	17.1%		1.10 dBμV
ACAM	18	2.23 dB	18.2%		1.29 dB
Two instruments					
TEV	74	1.93 dB	18.4%	3.96 dB	2.91 dB
ACP	74	1.78 dBμV	11.1%	1.89 dBμV	1.52 dBμV
AFR	74	2.28 dBμV	17.3%	4.38 dBμV	3.23 dBμV
PAR	36	1.14 dBμV	11.1%	5.42 dBμV	3.86 dBμV

* $q_{HFCT}$ in pC is the indicated instrument reading; see Section 2.1.

**Table 2:** Simulated noise sources and their representative substation equivalents.

Simulated Noise Source	Coupling Pathway	Substation Equivalent
Phase-Controlled AC Load Controller (Phase-cut AC)	Conducted, radiated EM	Legacy thyristor-based (SCR) station battery chargers; phase-angle controlled HVAC enclosure heaters; older solid-state lighting dimmers in control rooms.
High-Frequency Switched-Mode Power Supply (SMPS)	Conducted, radiated EM, structure-borne acoustic	Modern DIN-rail auxiliary power supplies; DC-DC converters inside digital protection relays (IEDs); SCADA RTU power modules; modern switch-mode battery chargers.
Electronic LED Driver	Conducted, radiated EM	Indoor substation building or control room’s LED lighting fixtures; electronic ballasts from legacy fluorescent lighting.
Narrowband Ultrasonic Source	Airborne and structure-borne acoustic	Small air leaks in pneumatic circuit breakers or compressed gas systems.
External Corona Discharge Source (Plasma ball)	Conducted, radiated EM, airborne acoustic	Brush sparking in cooling fan motors and auxiliary drives; arcing at loose or corroded connections; contact bounce in electromechanical relays and contactors

**Table 3:** Clean-condition detection of each PD source across PDIV and higher voltage steps.

Voltage Step	PAR (dBµV) BL = 1	ACAM (dB) BL = 1	AB (dBµV) BL = 2	ACP (dBµV) BL = 1	AFR (dBµV) BL = 1	TEV (dB) BL = 6	UWS-R (mV) BL = 9.5	UWS-A (mV) BL = 10.3	HFCT ($q_{HFCT}$/pC) BL = 47
Surface Discharge									
PDIV (1.8 kV)	24.0	24.0	18.0	4.5	9.8	20.0	24	265	307
1.5×PDIV (2.75 kV)	31.0	33.0	23.0	7.0	16.8	34.0	30	515	480
2×PDIV (3.66 kV)	36.0	39.0	27.0	10.0	21.2	38.5	34	601	860
3×PDIV (5.4 kV)	40.0	46.0	35.0	15.0	29.0	42.0	46	688	2014
Floating Discharge									
PDIV (7.75 kV)	28.5	29.0	17.0	4.0	12.8	33.5	87	716	1300
1.5×PDIV (12 kV)	33.5	35.0	26.0	7.5	19.5	45.0	117	960	3952
Corona Discharge									
PDIV (7.7 kV)	41.5	38.0	28.0	9.0	22.2	12.5	62	177	110
1.2×PDIV (9.2 kV)	48.5	51.0	41.0	23.5	35.5	47.0	220	210	690
Internal Discharge									
PDIV (3 kV)	19.0	27.0	11.0	1.0	1.5	27.0	41	616	290
1.25×PDIV (3.75 kV)	24.2	32.5	17.0	1.0	9.2	34.0	44	682	568
1.5×PDIV (4.5 kV)	29.0	36.5	22.0	4.0	14.2	35.0	61	710	654

BL is the quiescent baseline of each sensor, measured with the switchgear energized and no discharge or interference source active. Cell shading follows the reading magnitude, from pale yellow at the lower limit to dark red at the upper limit: a common linear scale from 1.0 to 51.0 for PAR, ACAM, AB, ACP, AFR and TEV, and a common logarithmic scale from 24 to 3952 for UWS-R, UWS-A and HFCT ($q_{HFCT}$ is the indicated instrument reading (Section 2.1)).

**Table 4:** Sensor responses to all four PD sources under each superimposed noise source.

Noise Condition	AB (dBµV) BL = 2	ACP (dBµV) BL = 1	AFR (dBµV) BL = 1	TEV (dB) BL = 6	UWS-A (mV) BL = 10.3	HFCT ($q_{HFCT}$/pC) BL = 47
Surface Discharge						
PDIV	18.0	4.5	9.8	20.0	265	307
+Phase-cut AC	19.0	10.0	11.0	33.0	283	696
+SMPS	29.0	8.5	16.0	32.0	310	974
+LED Driver	20.0	5.5	13.0	34.5	275	574
+NB Ultrasonic	63.0	41.0	53.0	32.5	290	804
+Plasma Ball	25.0	5.5	12.8	31.5	325	974
Floating Discharge						
PDIV	17.0	4.0	12.8	33.5	716	1300
+Phase-cut AC	18.0	4.0	15.2	38.0	720	1700
+SMPS	24.0	4.0	17.2	39.0	725	1800
+LED Driver	18.0	4.5	13.8	37.0	736	1450
+NB Ultrasonic	66.0	42.0	51.5	37.5	718	1350
+Plasma Ball	20.0	5.5	15.0	41.5	718	1650
Corona Discharge						
PDIV	28.0	9.0	22.2	12.5	177	110
+Phase-cut AC	32.0	11.5	26.8	29.0	194	611
+SMPS	32.0	11.0	27.2	16.0	185	850
+LED Driver	31.0	11.5	25.0	19.0	200	452
+NB Ultrasonic	63.0	45.5	54.0	15.5	180	119
+Plasma Ball	30.0	11.5	22.8	13.0	210	963
Internal Discharge						
PDIV	11.0	1.0	1.5	27.0	616	290
+Phase-cut AC	11.0	1.0	1.5	34.0	620	628
+SMPS	12.0	3.5	7.0	32.0	630	850
+LED Driver	12.0	1.0	3.5	33.0	625	400
+NB Ultrasonic	63.0	44.5	53.5	30.0	619	410
+Plasma Ball	15.0	4.0	4.0	29.0	622	876

BL is the quiescent baseline of each sensor, measured with the switchgear energized and no discharge or interference source active. All readings were taken at PDIV with the front panel closed. Cell shading follows the reading magnitude, from pale yellow at the lower limit to dark red at the upper limit: a common linear scale from 1.0 to 66.0 for AB, ACP, AFR and TEV, and a common logarithmic scale from 110 to 1800 for UWS-A and HFCT ($q_{HFCT}$ values are indicated instrument readings (Section 2.1)).

**Table 5:** Sensor performance matrix, in units of the standard uncertainty u of Table 1. Detection is (reading at PDIV—quiescent baseline)/u. Immunity is the worst case of (reading with noise—clean reading at PDIV)/u across the four discharge geometries. All values are dimensionless ratios. Boundaries of 1, 3 and 10 were adopted: a margin below 3 standard uncertainties is not reliably distinguishable from the baseline, while a margin of 10 or more allows the reading to be treated as quantitative. Detection: blind < 1, weak 1 to 3, moderate 3 to 10, good ≥ 10. The same boundaries apply to immunity, in the opposite sense: good under 3, moderate 3 to 10, noise-dominated over-read ≥ 10.

Sensor	Detection at PDIV				Noise Immunity				
	Surface	Floating	Corona	Internal	Phase-Cut AC	SMPS	LED Driver	NB Ultrasonic	Plasma Ball
HFCT	8.5	41.1	2.1	8.0	16.4	24.3	11.2	16.3	28.0
TEV	4.8	9.5	2.2	7.2	5.7	4.1	5.0	4.3	4.0
UWS-A	17.9	49.7	11.7	42.7	1.3	3.2	1.6	1.8	4.2
AB	14.5	13.6	23.6	8.2	3.6	10.0	2.7	47.3	6.4
ACP	2.3	2.0	5.3	0.0	3.6	2.6	1.6	28.6	2.0
AFR	2.7	3.7	6.6	0.2	1.4	1.9	1.0	16.1	0.9

Green: good (detection ≥ 10; immunity < 3); yellow: moderate (3–10); red: weak detection (1–3) or over-read immunity (≥ 10); grey: blind (< 1).

**Table 6:** Sensitivity and specificity (%) against detection threshold k·u above the quiescent baseline, from 24 conditions with a discharge present and 5 noise-only conditions. In the combined rules, “or” requires at least one named channel to respond and “and” requires both sides.

Decision Rule	k = 1		k = 2		k = 3		k = 4		k = 5	
	Sens.	Spec.	Sens.	Spec.	Sens.	Spec.	Sens.	Spec.	Sens.	Spec.
**Single channel**										
HFCT	100	0	100	20	92	20	92	20	92	20
TEV	100	40	100	80	92	80	83	80	75	100
UWS-A	100	60	100	60	100	100	100	100	100	100
AB	100	0	100	0	100	20	100	20	100	40
ACP	88	40	67	80	46	80	46	80	42	80
AFR	83	60	79	80	75	80	54	80	42	80
**Combined rules**										
HFCT or TEV or UWS-A	100	0	100	20	100	20	100	20	100	20
AB or ACP or AFR	100	0	100	0	100	20	100	20	100	40
(HFCT or TEV or UWS-A) and (AB or ACP or AFR)	100	0	100	20	100	40	100	40	100	60
(TEV or UWS-A) and (AB or ACP or AFR)	100	20	100	40	100	100	100	100	100	100

## Data Availability

The data presented in this study are contained within the article.
